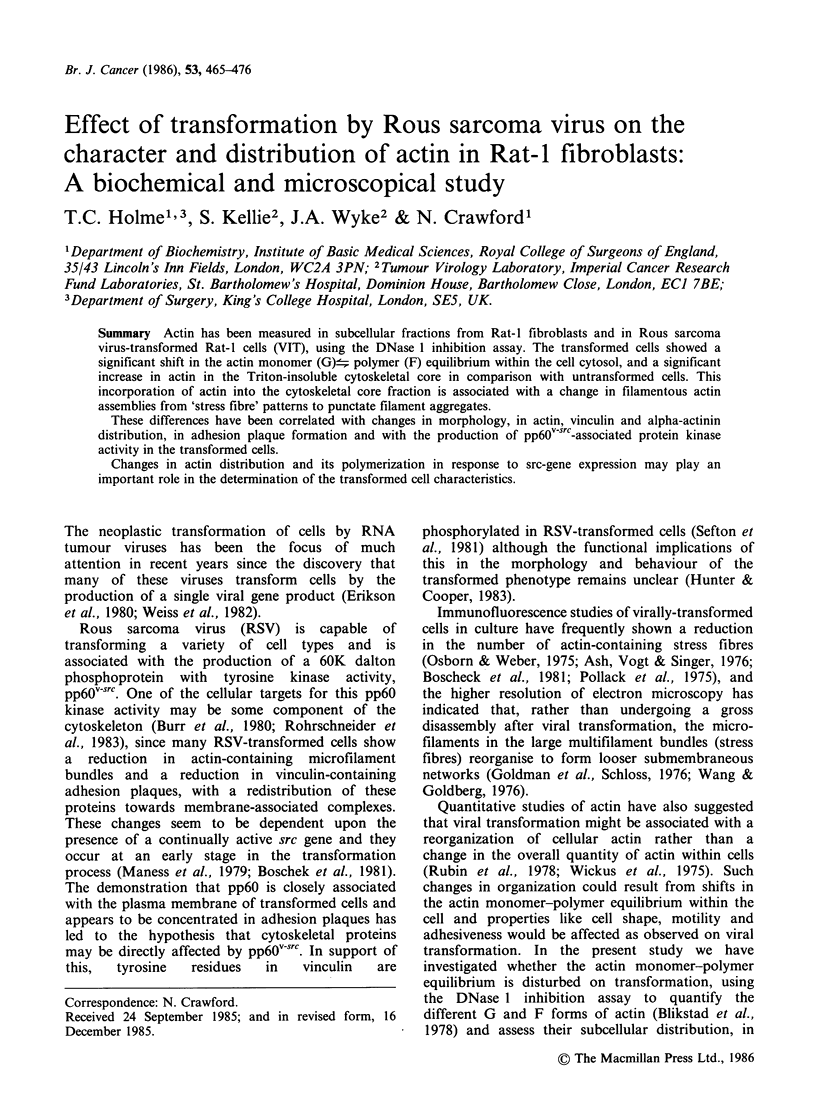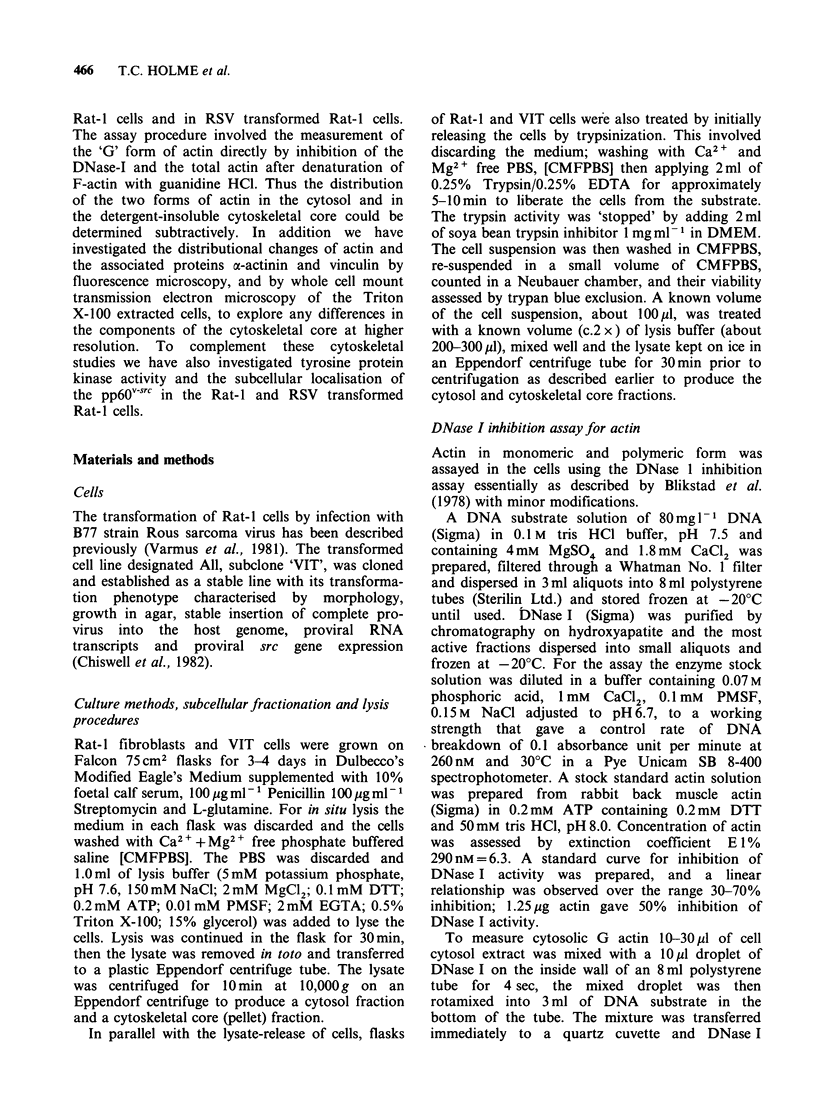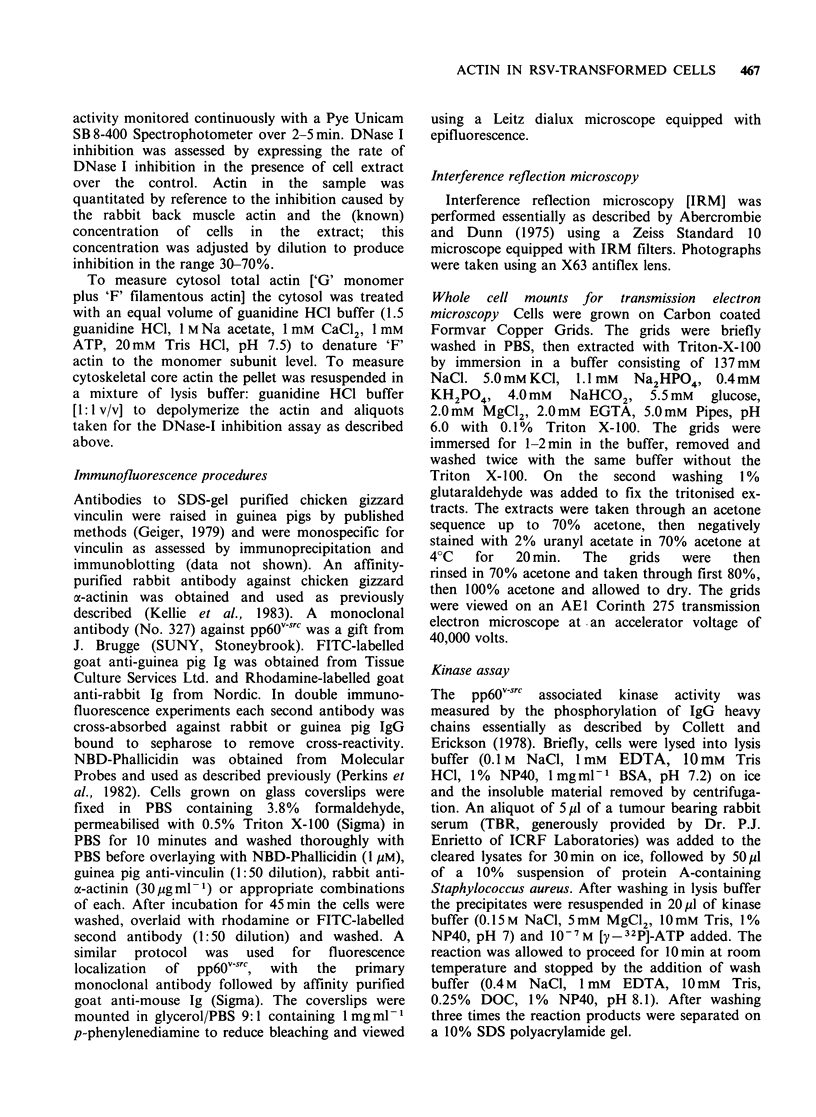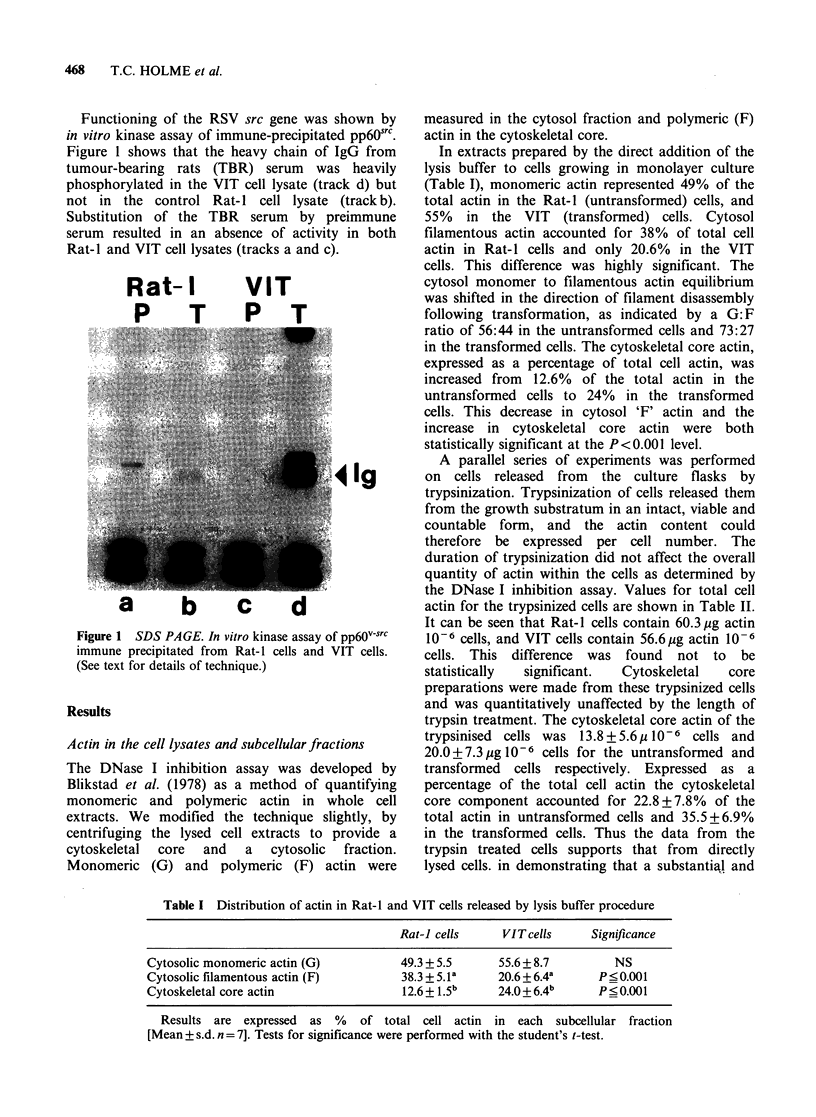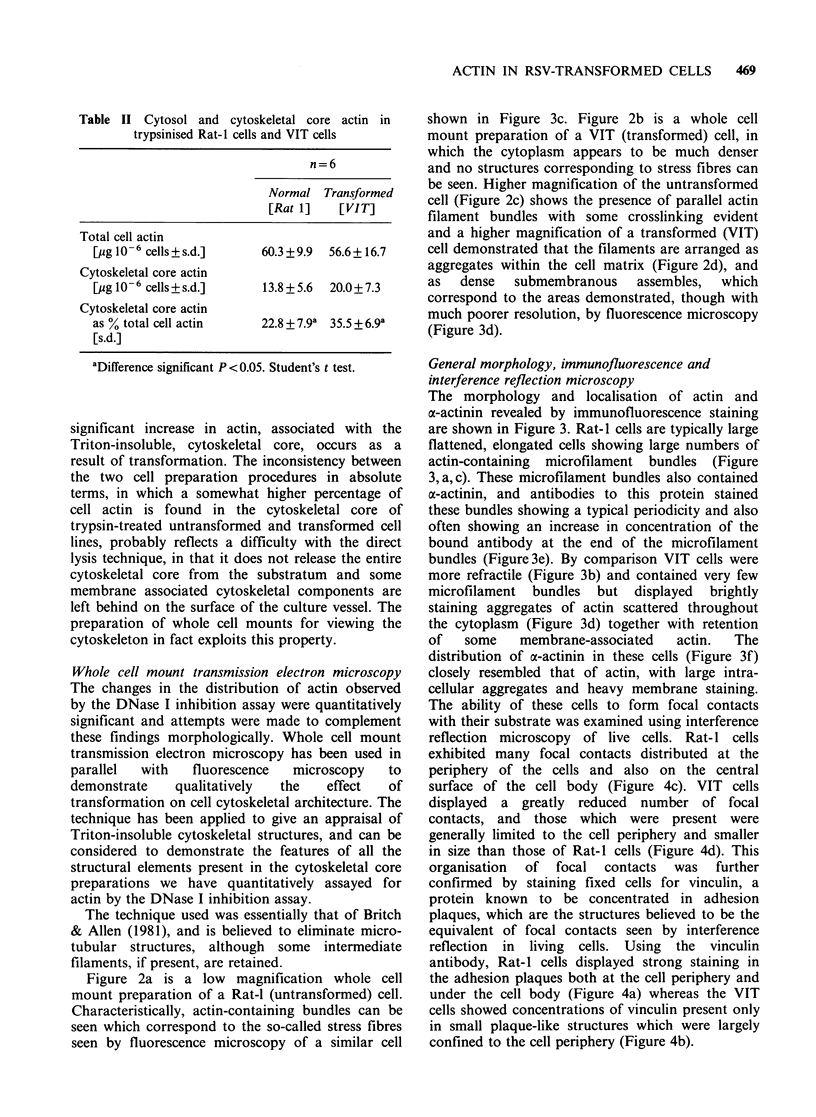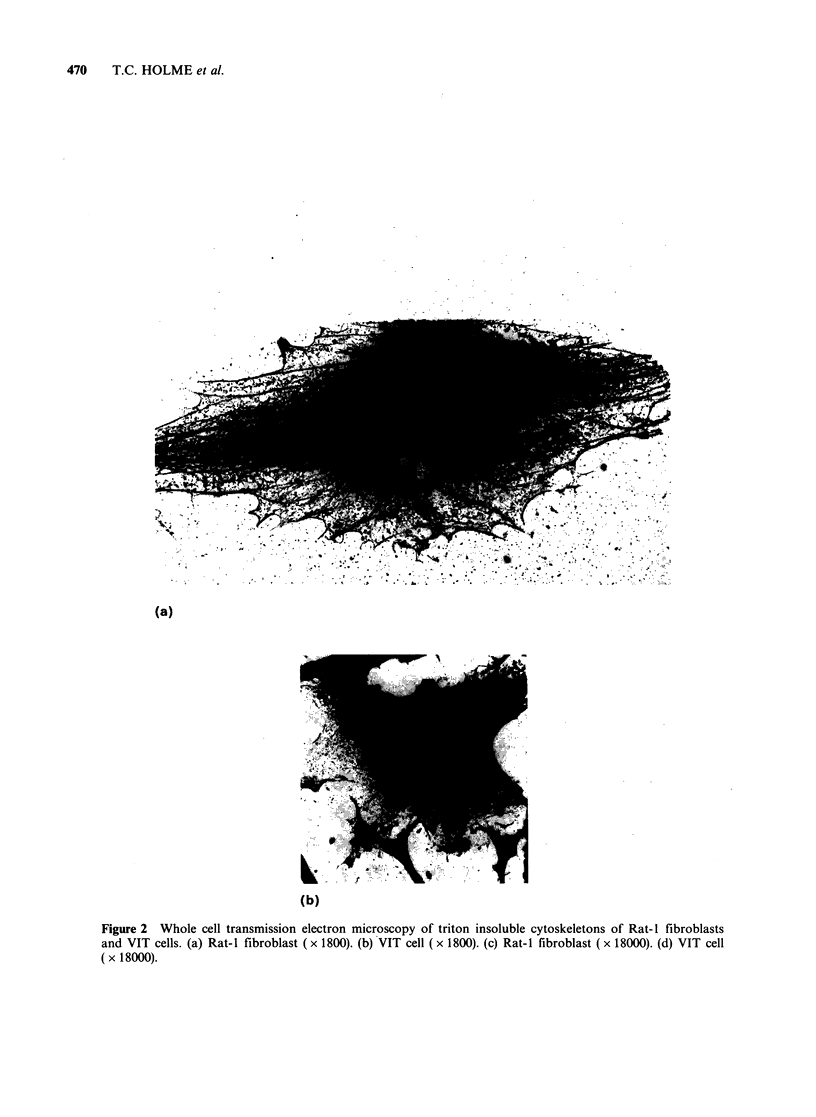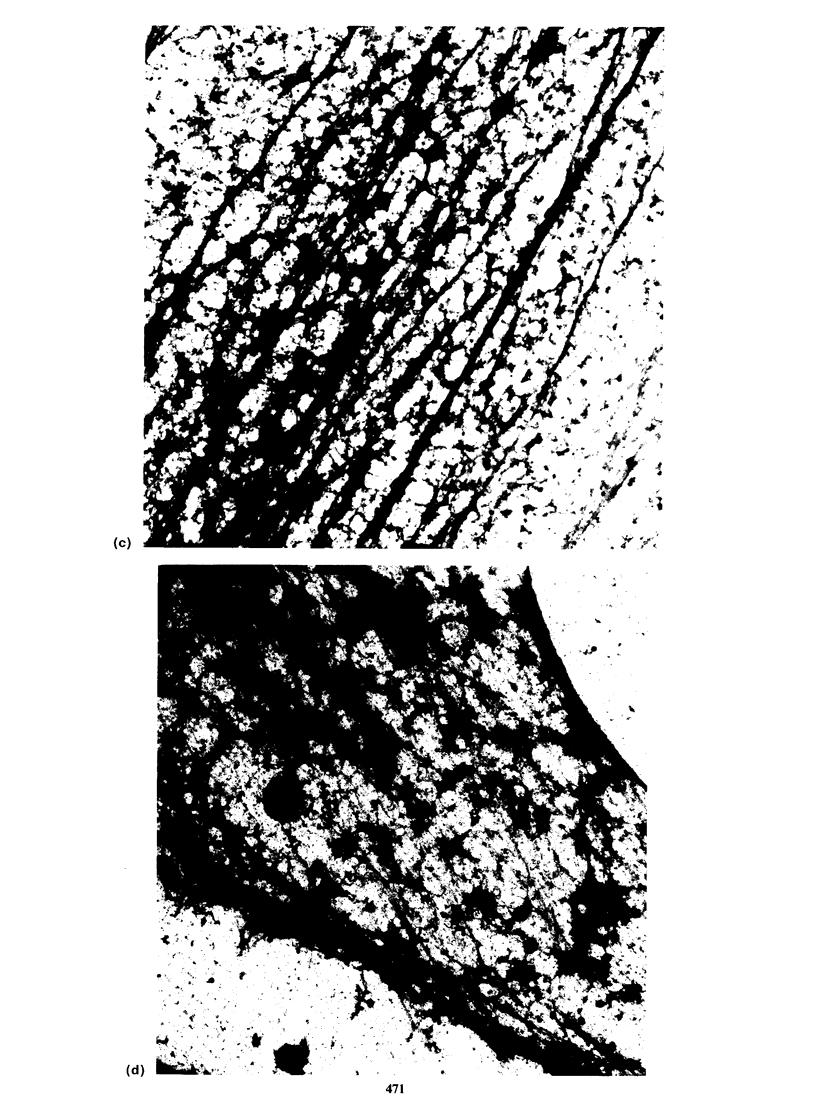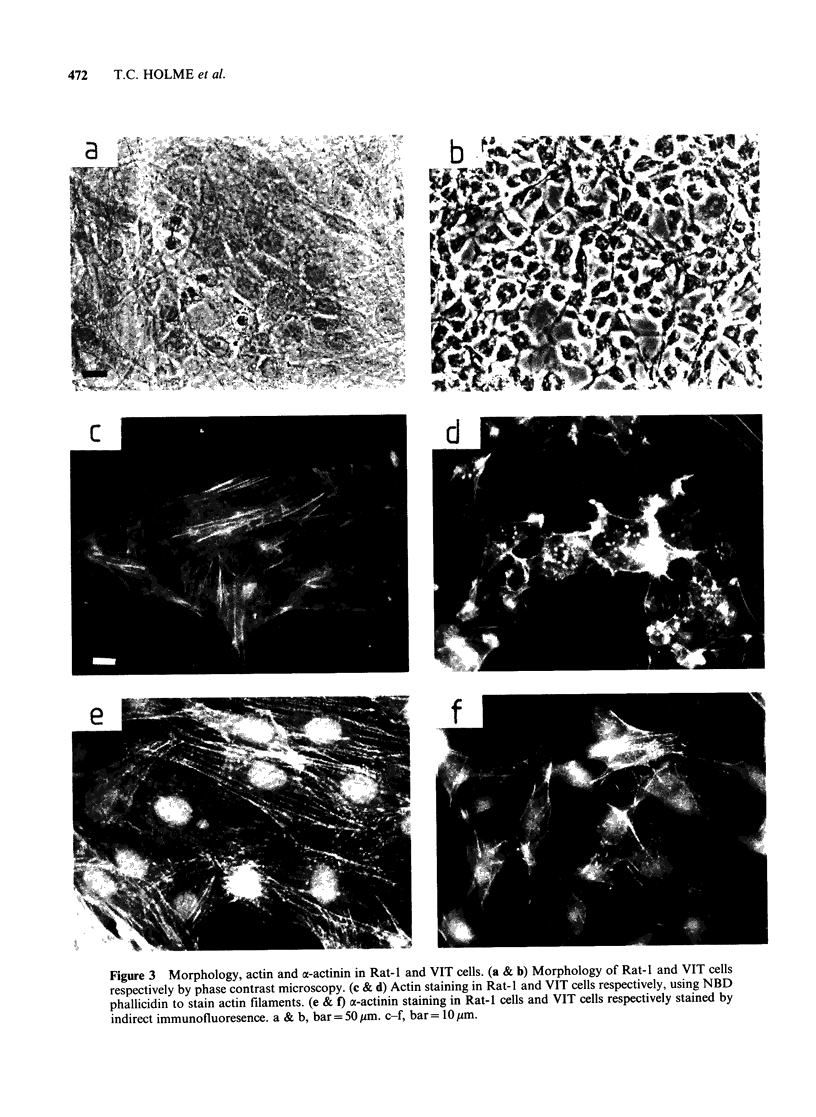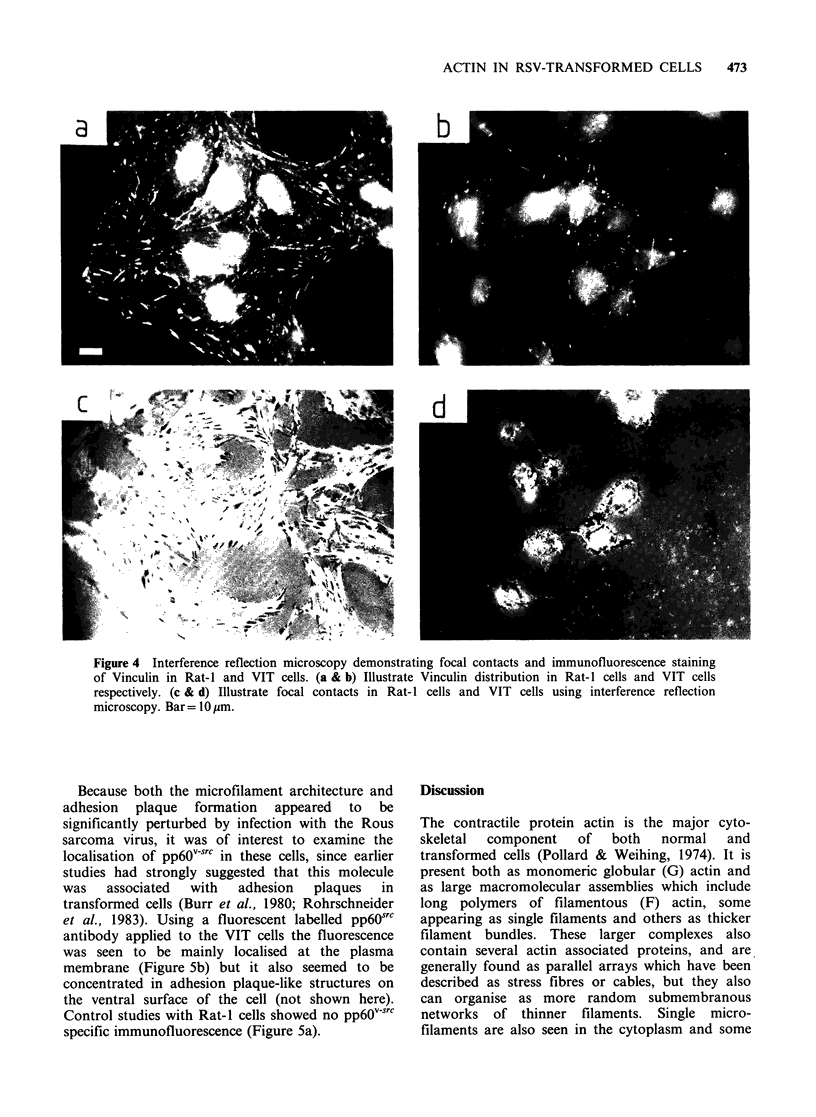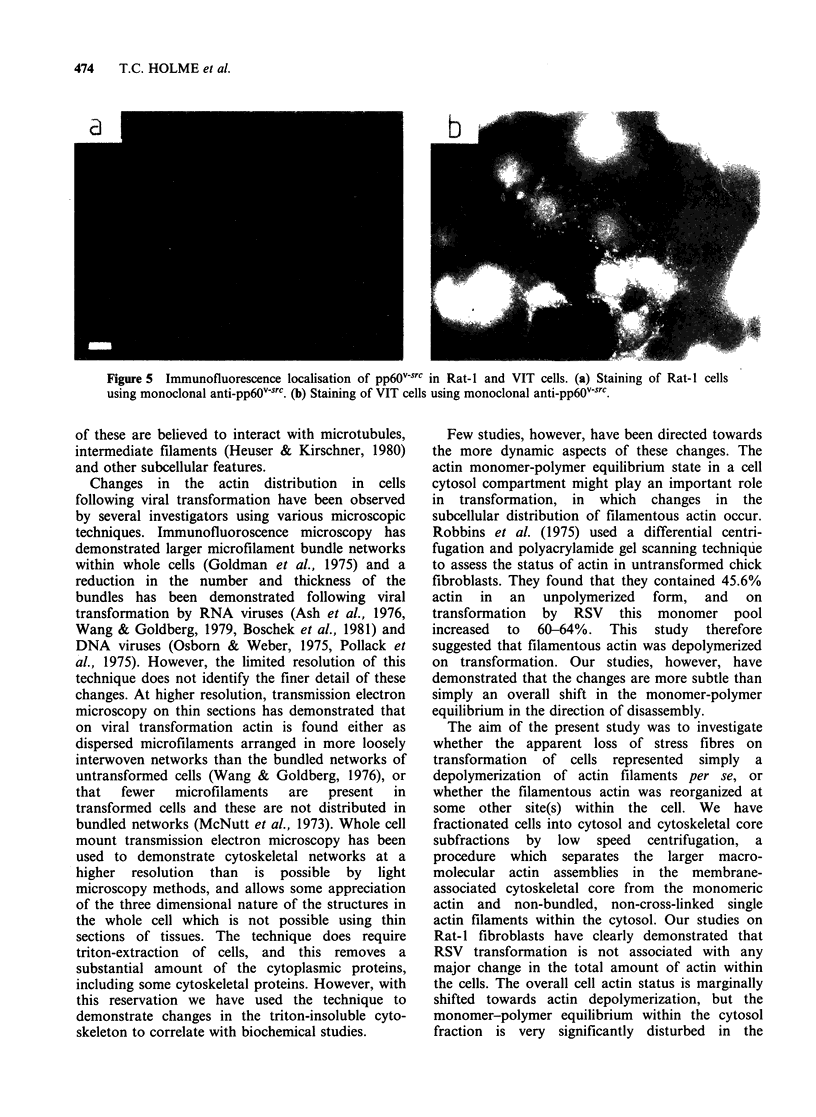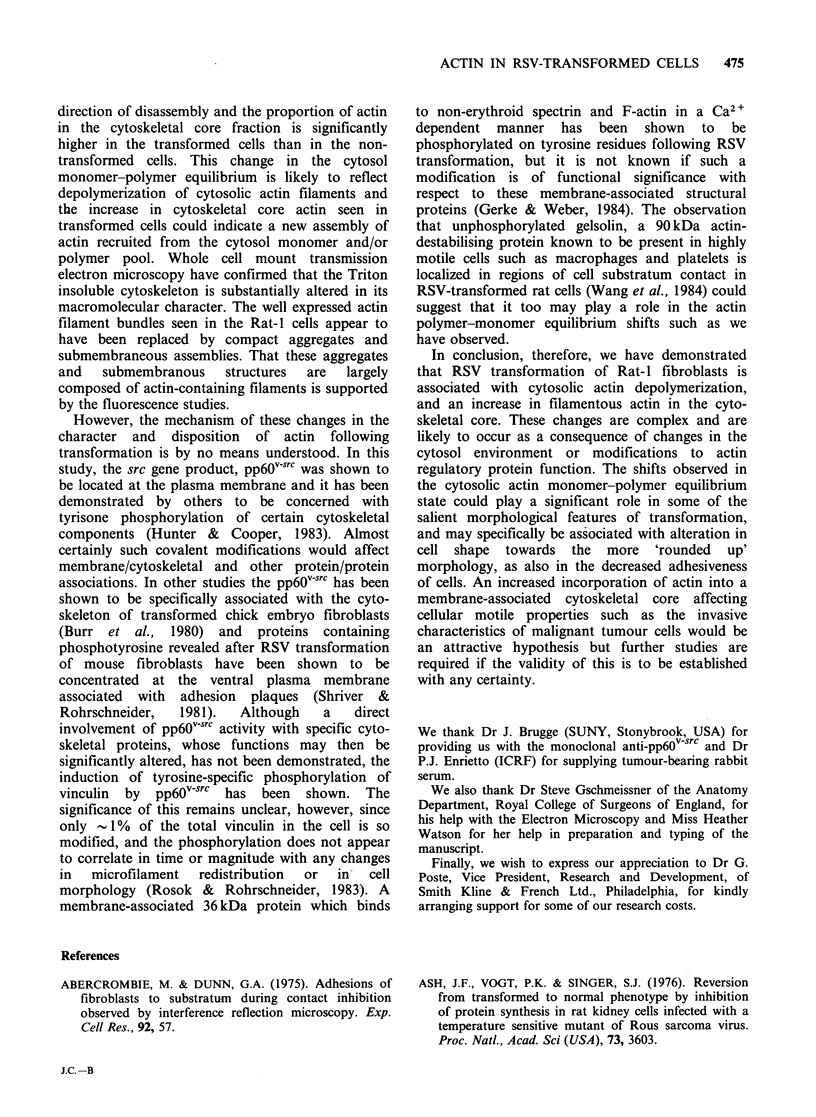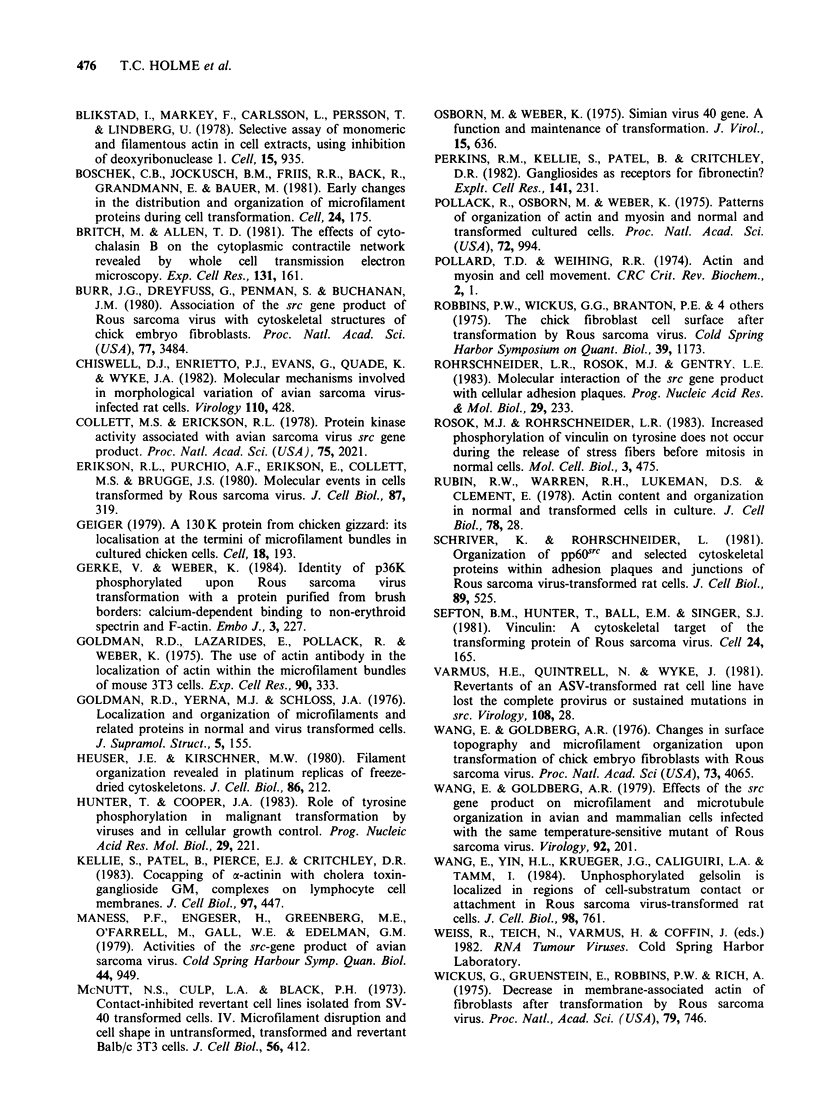# Effect of transformation by Rous sarcoma virus on the character and distribution of actin in Rat-1 fibroblasts: a biochemical and microscopical study.

**DOI:** 10.1038/bjc.1986.74

**Published:** 1986-04

**Authors:** T. C. Holme, S. Kellie, J. A. Wyke, N. Crawford

## Abstract

**Images:**


Br. J. Cancer (1986), 53, 465–476

### Effect of transformation by Rous sarcoma virus on the character and distribution of actin in Rat-1 fibroblasts: A biochemical and microscopical study

T.C. Holme[1,3], S. Kellie[2], J.A. Wyke[2] & N. Crawford[1]

[1]*Department of Biochemistry, Institute of Basic Medical Sciences, Royal College of Surgeons of England, 35/43 Lincoln's Inn Fields, London, WC2A 3PN;* [2]*Tumour Virology Laboratory, Imperial Cancer Research Fund Laboratories, St. Bartholomew's Hospital, Dominion House, Bartholomew Close, London, EC1 7BE;* [3]*Department of Surgery, King's College Hospital, London, SE5, UK.*

**Summary** Actin has been measured in subcellular fractions from Rat-1 fibroblasts and in Rous sarcoma virus-transformed Rat-1 cells (VIT), using the DNase 1 inhibition assay. The transformed cells showed a significant shift in the actin monomer (G)⇋ polymer (F) equilibrium within the cell cytosol, and a significant increase in actin in the Triton-insoluble cytoskeletal core in comparison with untransformed cells. This incorporation of actin into the cytoskeletal core fraction is associated with a change in filamentous actin assemblies from 'stress fibre' patterns to punctate filament aggregates.

These differences have been correlated with changes in morphology, in actin, vinculin and alpha-actinin distribution, in adhesion plaque formation and with the production of pp60$^{v\text{-}src}$-associated protein kinase activity in the transformed cells.

Changes in actin distribution and its polymerization in response to src-gene expression may play an important role in the determination of the transformed cell characteristics.

The neoplastic transformation of cells by RNA tumour viruses has been the focus of much attention in recent years since the discovery that many of these viruses transform cells by the production of a single viral gene product (Erikson *et al.*, 1980; Weiss *et al.*, 1982).

Rous sarcoma virus (RSV) is capable of transforming a variety of cell types and is associated with the production of a 60K dalton phosphoprotein with tyrosine kinase activity, pp60$^{v\text{-}src}$. One of the cellular targets for this pp60 kinase activity may be some component of the cytoskeleton (Burr *et al.*, 1980; Rohrschneider *et al.*, 1983), since many RSV-transformed cells show a reduction in actin-containing microfilament bundles and a reduction in vinculin-containing adhesion plaques, with a redistribution of these proteins towards membrane-associated complexes. These changes seem to be dependent upon the presence of a continually active *src* gene and they occur at an early stage in the transformation process (Maness *et al.*, 1979; Boschek *et al.*, 1981). The demonstration that pp60 is closely associated with the plasma membrane of transformed cells and appears to be concentrated in adhesion plaques has led to the hypothesis that cytoskeletal proteins may be directly affected by pp60$^{v\text{-}src}$. In support of this, tyrosine residues in vinculin are phosphorylated in RSV-transformed cells (Sefton *et al.*, 1981) although the functional implications of this in the morphology and behaviour of the transformed phenotype remains unclear (Hunter & Cooper, 1983).

Immunofluorescence studies of virally-transformed cells in culture have frequently shown a reduction in the number of actin-containing stress fibres (Osborn & Weber, 1975; Ash, Vogt & Singer, 1976; Boscheck *et al.*, 1981; Pollack *et al.*, 1975), and the higher resolution of electron microscopy has indicated that, rather than undergoing a gross disassembly after viral transformation, the microfilaments in the large multifilament bundles (stress fibres) reorganise to form looser submembraneous networks (Goldman *et al.*, Schloss, 1976; Wang & Goldberg, 1976).

Quantitative studies of actin have also suggested that viral transformation might be associated with a reorganization of cellular actin rather than a change in the overall quantity of actin within cells (Rubin *et al.*, 1978; Wickus *et al.*, 1975). Such changes in organization could result from shifts in the actin monomer–polymer equilibrium within the cell and properties like cell shape, motility and adhesiveness would be affected as observed on viral transformation. In the present study we have investigated whether the actin monomer–polymer equilibrium is disturbed on transformation, using the DNase 1 inhibition assay to quantify the different G and F forms of actin (Blikstad *et al.*, 1978) and assess their subcellular distribution, in

Correspondence: N. Crawford.
Received 24 September 1985; and in revised form, 16 December 1985.

© The Macmillan Press Ltd., 1986

Rat-1 cells and in RSV transformed Rat-1 cells. The assay procedure involved the measurement of the 'G' form of actin directly by inhibition of the DNase-I and the total actin after denaturation of F-actin with guanidine HCl. Thus the distribution of the two forms of actin in the cytosol and in the detergent-insoluble cytoskeletal core could be determined subtractively. In addition we have investigated the distributional changes of actin and the associated proteins α-actinin and vinculin by fluorescence microscopy, and by whole cell mount transmission electron microscopy of the Triton X-100 extracted cells, to explore any differences in the components of the cytoskeletal core at higher resolution. To complement these cytoskeletal studies we have also investigated tyrosine protein kinase activity and the subcellular localisation of the $pp60^{v\text{-}src}$ in the Rat-1 and RSV transformed Rat-1 cells.

### Materials and methods

### *Cells*

The transformation of Rat-1 cells by infection with B77 strain Rous sarcoma virus has been described previously (Varmus *et al.*, 1981). The transformed cell line designated All, subclone 'VIT', was cloned and established as a stable line with its transformation phenotype characterised by morphology, growth in agar, stable insertion of complete provirus into the host genome, proviral RNA transcripts and proviral *src* gene expression (Chiswell *et al.*, 1982).

### *Culture methods, subcellular fractionation and lysis procedures*

Rat-1 fibroblasts and VIT cells were grown on Falcon 75 $cm^2$ flasks for 3–4 days in Dulbecco's Modified Eagle's Medium supplemented with 10% foetal calf serum, 100 $\mu g\,ml^{-1}$ Penicillin 100 $\mu g\,ml^{-1}$ Streptomycin and L-glutamine. For *in situ* lysis the medium in each flask was discarded and the cells washed with $Ca^{2+}+Mg^{2+}$ free phosphate buffered saline [CMFPBS]. The PBS was discarded and 1.0 ml of lysis buffer (5 mM potassium phosphate, pH 7.6, 150 mM NaCl; 2 mM $MgCl_2$; 0.1 mM DTT; 0.2 mM ATP; 0.01 mM PMSF; 2 mM EGTA; 0.5% Triton X-100; 15% glycerol) was added to lyse the cells. Lysis was continued in the flask for 30 min, then the lysate was removed *in toto* and transferred to a plastic Eppendorf centrifuge tube. The lysate was centrifuged for 10 min at 10,000 *g* on an Eppendorf centrifuge to produce a cytosol fraction and a cytoskeletal core (pellet) fraction.

In parallel with the lysate-release of cells, flasks of Rat-1 and VIT cells were also treated by initially releasing the cells by trypsinization. This involved discarding the medium; washing with $Ca^{2+}$ and $Mg^{2+}$ free PBS, [CMFPBS] then applying 2 ml of 0.25% Trypsin/0.25% EDTA for approximately 5–10 min to liberate the cells from the substrate. The trypsin activity was 'stopped' by adding 2 ml of soya bean trypsin inhibitor 1 $mg\,ml^{-1}$ in DMEM. The cell suspension was then washed in CMFPBS, re-suspended in a small volume of CMFPBS, counted in a Neubauer chamber, and their viability assessed by trypan blue exclusion. A known volume of the cell suspension, about 100 $\mu l$, was treated with a known volume (c.2 ×) of lysis buffer (about 200–300 $\mu l$), mixed well and the lysate kept on ice in an Eppendorf centrifuge tube for 30 min prior to centrifugation as described earlier to produce the cytosol and cytoskeletal core fractions.

### *DNase I inhibition assay for actin*

Actin in monomeric and polymeric form was assayed in the cells using the DNase 1 inhibition assay essentially as described by Blikstad *et al.* (1978) with minor modifications.

A DNA substrate solution of 80 $mg\,l^{-1}$ DNA (Sigma) in 0.1 M tris HCl buffer, pH 7.5 and containing 4 mM $MgSO_4$ and 1.8 mM $CaCl_2$ was prepared, filtered through a Whatman No. 1 filter and dispersed in 3 ml aliquots into 8 ml polystyrene tubes (Sterilin Ltd.) and stored frozen at −20°C until used. DNase I (Sigma) was purified by chromatography on hydroxyapatite and the most active fractions dispersed into small aliquots and frozen at −20°C. For the assay the enzyme stock solution was diluted in a buffer containing 0.07 M phosphoric acid, 1 mM $CaCl_2$, 0.1 mM PMSF, 0.15 M NaCl adjusted to pH 6.7, to a working strength that gave a control rate of DNA breakdown of 0.1 absorbance unit per minute at 260 nM and 30°C in a Pye Unicam SB 8-400 spectrophotometer. A stock standard actin solution was prepared from rabbit back muscle actin (Sigma) in 0.2 mM ATP containing 0.2 mM DTT and 50 mM tris HCl, pH 8.0. Concentration of actin was assessed by extinction coefficient E 1% 290 nM = 6.3. A standard curve for inhibition of DNase I activity was prepared, and a linear relationship was observed over the range 30–70% inhibition; 1.25 $\mu g$ actin gave 50% inhibition of DNase I activity.

To measure cytosolic G actin 10–30 $\mu l$ of cell cytosol extract was mixed with a 10 $\mu l$ droplet of DNase I on the inside wall of an 8 ml polystyrene tube for 4 sec, the mixed droplet was then rotamixed into 3 ml of DNA substrate in the bottom of the tube. The mixture was transferred immediately to a quartz cuvette and DNase I

activity monitored continuously with a Pye Unicam SB 8-400 Spectrophotometer over 2–5 min. DNase I inhibition was assessed by expressing the rate of DNase I inhibition in the presence of cell extract over the control. Actin in the sample was quantitated by reference to the inhibition caused by the rabbit back muscle actin and the (known) concentration of cells in the extract; this concentration was adjusted by dilution to produce inhibition in the range 30–70%.

To measure cytosol total actin ['G' monomer plus 'F' filamentous actin] the cytosol was treated with an equal volume of guanidine HCl buffer (1.5 guanidine HCl, 1 M Na acetate, 1 mM $CaCl_2$, 1 mM ATP, 20 mM Tris HCl, pH 7.5) to denature 'F' actin to the monomer subunit level. To measure cytoskeletal core actin the pellet was resuspended in a mixture of lysis buffer: guanidine HCl buffer [1:1 v/v] to depolymerize the actin and aliquots taken for the DNase-I inhibition assay as described above.

*Immunofluorescence procedures*

Antibodies to SDS-gel purified chicken gizzard vinculin were raised in guinea pigs by published methods (Geiger, 1979) and were monospecific for vinculin as assessed by immunoprecipitation and immunoblotting (data not shown). An affinity-purified rabbit antibody against chicken gizzard α-actinin was obtained and used as previously described (Kellie *et al.*, 1983). A monoclonal antibody (No. 327) against $pp60^{v\text{-}src}$ was a gift from J. Brugge (SUNY, Stoneybrook). FITC-labelled goat anti-guinea pig Ig was obtained from Tissue Culture Services Ltd. and Rhodamine-labelled goat anti-rabbit Ig from Nordic. In double immunofluorescence experiments each second antibody was cross-absorbed against rabbit or guinea pig IgG bound to sepharose to remove cross-reactivity. NBD-Phallicidin was obtained from Molecular Probes and used as described previously (Perkins *et al.*, 1982). Cells grown on glass coverslips were fixed in PBS containing 3.8% formaldehyde, permeabilised with 0.5% Triton X-100 (Sigma) in PBS for 10 minutes and washed thoroughly with PBS before overlaying with NBD-Phallicidin (1 μM), guinea pig anti-vinculin (1:50 dilution), rabbit anti-α-actinin (30 $\mu g\,ml^{-1}$) or appropriate combinations of each. After incubation for 45 min the cells were washed, overlaid with rhodamine or FITC-labelled second antibody (1:50 dilution) and washed. A similar protocol was used for fluorescence localization of $pp60^{v\text{-}src}$, with the primary monoclonal antibody followed by affinity purified goat anti-mouse Ig (Sigma). The coverslips were mounted in glycerol/PBS 9:1 containing 1 $mg\,ml^{-1}$ *p*-phenylenediamine to reduce bleaching and viewed using a Leitz dialux microscope equipped with epifluorescence.

*Interference reflection microscopy*

Interference reflection microscopy [IRM] was performed essentially as described by Abercrombie and Dunn (1975) using a Zeiss Standard 10 microscope equipped with IRM filters. Photographs were taken using an X63 antiflex lens.

*Whole cell mounts for transmission electron microscopy* Cells were grown on Carbon coated Formvar Copper Grids. The grids were briefly washed in PBS, then extracted with Triton-X-100 by immersion in a buffer consisting of 137 mM NaCl. 5.0 mM KCl, 1.1 mM $Na_2HPO_4$, 0.4 mM $KH_2PO_4$, 4.0 mM $NaHCO_2$, 5.5 mM glucose, 2.0 mM $MgCl_2$, 2.0 mM EGTA, 5.0 mM Pipes, pH 6.0 with 0.1% Triton X-100. The grids were immersed for 1–2 min in the buffer, removed and washed twice with the same buffer without the Triton X-100. On the second washing 1% glutaraldehyde was added to fix the tritonised extracts. The extracts were taken through an acetone sequence up to 70% acetone, then negatively stained with 2% uranyl acetate in 70% acetone at 4°C for 20 min. The grids were then rinsed in 70% acetone and taken through first 80%, then 100% acetone and allowed to dry. The grids were viewed on an AE1 Corinth 275 transmission electron microscope at an accelerator voltage of 40,000 volts.

*Kinase assay*

The $pp60^{v\text{-}src}$ associated kinase activity was measured by the phosphorylation of IgG heavy chains essentially as described by Collett and Erickson (1978). Briefly, cells were lysed into lysis buffer (0.1 M NaCl, 1 mM EDTA, 10 mM Tris HCl, 1% NP40, 1 $mg\,ml^{-1}$ BSA, pH 7.2) on ice and the insoluble material removed by centrifugation. An aliquot of 5 μl of a tumour bearing rabbit serum (TBR, generously provided by Dr. P.J. Enrietto of ICRF Laboratories) was added to the cleared lysates for 30 min on ice, followed by 50 μl of a 10% suspension of protein A-containing *Staphylococcus aureus*. After washing in lysis buffer the precipitates were resuspended in 20 μl of kinase buffer (0.15 M NaCl, 5 mM $MgCl_2$, 10 mM Tris, 1% NP40, pH 7) and $10^{-7}$ M [$\gamma-^{32}P$]-ATP added. The reaction was allowed to proceed for 10 min at room temperature and stopped by the addition of wash buffer (0.4 M NaCl, 1 mM EDTA, 10 mM Tris, 0.25% DOC, 1% NP40, pH 8.1). After washing three times the reaction products were separated on a 10% SDS polyacrylamide gel.

Functioning of the RSV *src* gene was shown by *in vitro* kinase assay of immune-precipitated $pp60^{src}$. Figure 1 shows that the heavy chain of IgG from tumour-bearing rats (TBR) serum was heavily phosphorylated in the VIT cell lysate (track d) but not in the control Rat-1 cell lysate (track b). Substitution of the TBR serum by preimmune serum resulted in an absence of activity in both Rat-1 and VIT cell lysates (tracks a and c).

**Figure 1** *SDS PAGE. In vitro* kinase assay of $pp60^{v\text{-}src}$ immune precipitated from Rat-1 cells and VIT cells. (See text for details of technique.)

### Results

### *Actin in the cell lysates and subcellular fractions*

The DNase I inhibition assay was developed by Blikstad *et al.* (1978) as a method of quantifying monomeric and polymeric actin in whole cell extracts. We modified the technique slightly, by centrifuging the lysed cell extracts to provide a cytoskeletal core and a cytosolic fraction. Monomeric (G) and polymeric (F) actin were measured in the cytosol fraction and polymeric (F) actin in the cytoskeletal core.

In extracts prepared by the direct addition of the lysis buffer to cells growing in monolayer culture (Table I), monomeric actin represented 49% of the total actin in the Rat-1 (untransformed) cells, and 55% in the VIT (transformed) cells. Cytosol filamentous actin accounted for 38% of total cell actin in Rat-1 cells and only 20.6% in the VIT cells. This difference was highly significant. The cytosol monomer to filamentous actin equilibrium was shifted in the direction of filament disassembly following transformation, as indicated by a G:F ratio of 56:44 in the untransformed cells and 73:27 in the transformed cells. The cytoskeletal core actin, expressed as a percentage of total cell actin, was increased from 12.6% of the total actin in the untransformed cells to 24% in the transformed cells. This decrease in cytosol 'F' actin and the increase in cytoskeletal core actin were both statistically significant at the $P<0.001$ level.

A parallel series of experiments was performed on cells released from the culture flasks by trypsinization. Trypsinization of cells released them from the growth substratum in an intact, viable and countable form, and the actin content could therefore be expressed per cell number. The duration of trypsinization did not affect the overall quantity of actin within the cells as determined by the DNase I inhibition assay. Values for total cell actin for the trypsinized cells are shown in Table II. It can be seen that Rat-1 cells contain 60.3 $\mu$g actin $10^{-6}$ cells, and VIT cells contain 56.6 $\mu$g actin $10^{-6}$ cells. This difference was found not to be statistically significant. Cytoskeletal core preparations were made from these trypsinized cells and was quantitatively unaffected by the length of trypsin treatment. The cytoskeletal core actin of the trypsinised cells was $13.8 \pm 5.6\,\mu\,10^{-6}$ cells and $20.0 \pm 7.3\,\mu g\,10^{-6}$ cells for the untransformed and transformed cells respectively. Expressed as a percentage of the total cell actin the cytoskeletal core component accounted for $22.8 \pm 7.8\%$ of the total actin in untransformed cells and $35.5 \pm 6.9\%$ in the transformed cells. Thus the data from the trypsin treated cells supports that from directly lysed cells. in demonstrating that a substantial and

**Table I** Distribution of actin in Rat-1 and VIT cells released by lysis buffer procedure

| | *Rat-1 cells* | *VIT cells* | *Significance* |
|---|---|---|---|
| Cytosolic monomeric actin (G) | $49.3 \pm 5.5$ | $55.6 \pm 8.7$ | NS |
| Cytosolic filamentous actin (F) | $38.3 \pm 5.1^a$ | $20.6 \pm 6.4^a$ | $P \leq 0.001$ |
| Cytoskeletal core actin | $12.6 \pm 1.5^b$ | $24.0 \pm 6.4^b$ | $P \leq 0.001$ |

Results are expressed as % of total cell actin in each subcellular fraction [Mean $\pm$ s.d. $n=7$]. Tests for significance were performed with the student's $t$-test.

**Table II** Cytosol and cytoskeletal core actin in trypsinised Rat-1 cells and VIT cells

| | $n=6$ | |
|---|---|---|
| | *Normal* [*Rat* 1] | *Transformed* [*VIT*] |
| Total cell actin [$\mu$g $10^{-6}$ cells $\pm$ s.d.] | $60.3 \pm 9.9$ | $56.6 \pm 16.7$ |
| Cytoskeletal core actin [$\mu$g $10^{-6}$ cells $\pm$ s.d.] | $13.8 \pm 5.6$ | $20.0 \pm 7.3$ |
| Cytoskeletal core actin as % total cell actin [s.d.] | $22.8 \pm 7.9$[a] | $35.5 \pm 6.9$[a] |

[a]Difference significant $P<0.05$. Student's $t$ test.

significant increase in actin, associated with the Triton-insoluble, cytoskeletal core, occurs as a result of transformation. The inconsistency between the two cell preparation procedures in absolute terms, in which a somewhat higher percentage of cell actin is found in the cytoskeletal core of trypsin-treated untransformed and transformed cell lines, probably reflects a difficulty with the direct lysis technique, in that it does not release the entire cytoskeletal core from the substratum and some membrane associated cytoskeletal components are left behind on the surface of the culture vessel. The preparation of whole cell mounts for viewing the cytoskeleton in fact exploits this property.

*Whole cell mount transmission electron microscopy*

The changes in the distribution of actin observed by the DNase I inhibition assay were quantitatively significant and attempts were made to complement these findings morphologically. Whole cell mount transmission electron microscopy has been used in parallel with fluorescence microscopy to demonstrate qualitatively the effect of transformation on cell cytoskeletal architecture. The technique has been applied to give an appraisal of Triton-insoluble cytoskeletal structures, and can be considered to demonstrate the features of all the structural elements present in the cytoskeletal core preparations we have quantitatively assayed for actin by the DNase I inhibition assay.

The technique used was essentially that of Britch & Allen (1981), and is believed to eliminate microtubular structures, although some intermediate filaments, if present, are retained.

Figure 2a is a low magnification whole cell mount preparation of a Rat-l (untransformed) cell. Characteristically, actin-containing bundles can be seen which correspond to the so-called stress fibres seen by fluorescence microscopy of a similar cell shown in Figure 3c. Figure 2b is a whole cell mount preparation of a VIT (transformed) cell, in which the cytoplasm appears to be much denser and no structures corresponding to stress fibres can be seen. Higher magnification of the untransformed cell (Figure 2c) shows the presence of parallel actin filament bundles with some crosslinking evident and a higher magnification of a transformed (VIT) cell demonstrated that the filaments are arranged as aggregates within the cell matrix (Figure 2d), and as dense submembranous assembles, which correspond to the areas demonstrated, though with much poorer resolution, by fluorescence microscopy (Figure 3d).

*General morphology, immunofluorescence and interference reflection microscopy*

The morphology and localisation of actin and α-actinin revealed by immunofluorescence staining are shown in Figure 3. Rat-1 cells are typically large flattened, elongated cells showing large numbers of actin-containing microfilament bundles (Figure 3, a, c). These microfilament bundles also contained α-actinin, and antibodies to this protein stained these bundles showing a typical periodicity and also often showing an increase in concentration of the bound antibody at the end of the microfilament bundles (Figure 3e). By comparison VIT cells were more refractile (Figure 3b) and contained very few microfilament bundles but displayed brightly staining aggregates of actin scattered throughout the cytoplasm (Figure 3d) together with retention of some membrane-associated actin. The distribution of α-actinin in these cells (Figure 3f) closely resembled that of actin, with large intracellular aggregates and heavy membrane staining. The ability of these cells to form focal contacts with their substrate was examined using interference reflection microscopy of live cells. Rat-1 cells exhibited many focal contacts distributed at the periphery of the cells and also on the central surface of the cell body (Figure 4c). VIT cells displayed a greatly reduced number of focal contacts, and those which were present were generally limited to the cell periphery and smaller in size than those of Rat-1 cells (Figure 4d). This organisation of focal contacts was further confirmed by staining fixed cells for vinculin, a protein known to be concentrated in adhesion plaques, which are the structures believed to be the equivalent of focal contacts seen by interference reflection in living cells. Using the vinculin antibody, Rat-1 cells displayed strong staining in the adhesion plaques both at the cell periphery and under the cell body (Figure 4a) whereas the VIT cells showed concentrations of vinculin present only in small plaque-like structures which were largely confined to the cell periphery (Figure 4b).

(a)

(b)

**Figure 2** Whole cell transmission electron microscopy of triton insoluble cytoskeletons of Rat-1 fibroblasts and VIT cells. (a) Rat-1 fibroblast ( × 1800). (b) VIT cell ( × 1800). (c) Rat-1 fibroblast ( × 18000). (d) VIT cell ( × 18000).

(c)

(d)

**Figure 3** Morphology, actin and α-actinin in Rat-1 and VIT cells. (**a** & **b**) Morphology of Rat-1 and VIT cells respectively by phase contrast microscopy. (**c** & **d**) Actin staining in Rat-1 and VIT cells respectively, using NBD phallicidin to stain actin filaments. (**e** & **f**) α-actinin staining in Rat-1 cells and VIT cells respectively stained by indirect immunofluoresence. a & b, bar = 50 μm. c–f, bar = 10 μm.

**Figure 4** Interference reflection microscopy demonstrating focal contacts and immunofluorescence staining of Vinculin in Rat-1 and VIT cells. (**a & b**) Illustrate Vinculin distribution in Rat-1 cells and VIT cells respectively. (**c & d**) Illustrate focal contacts in Rat-1 cells and VIT cells using interference reflection microscopy. Bar = 10 $\mu$m.

Because both the microfilament architecture and adhesion plaque formation appeared to be significantly perturbed by infection with the Rous sarcoma virus, it was of interest to examine the localisation of pp60$^{v\text{-}src}$ in these cells, since earlier studies had strongly suggested that this molecule was associated with adhesion plaques in transformed cells (Burr *et al.*, 1980; Rohrschneider *et al.*, 1983). Using a fluorescent labelled pp60$^{src}$ antibody applied to the VIT cells the fluorescence was seen to be mainly localised at the plasma membrane (Figure 5b) but it also seemed to be concentrated in adhesion plaque-like structures on the ventral surface of the cell (not shown here). Control studies with Rat-1 cells showed no pp60$^{v\text{-}src}$ specific immunofluorescence (Figure 5a).

### Discussion

The contractile protein actin is the major cytoskeletal component of both normal and transformed cells (Pollard & Weihing, 1974). It is present both as monomeric globular (G) actin and as large macromolecular assemblies which include long polymers of filamentous (F) actin, some appearing as single filaments and others as thicker filament bundles. These larger complexes also contain several actin associated proteins, and are generally found as parallel arrays which have been described as stress fibres or cables, but they also can organise as more random submembranous networks of thinner filaments. Single microfilaments are also seen in the cytoplasm and some

**Figure 5** Immunofluorescence localisation of pp60$^{v\text{-}src}$ in Rat-1 and VIT cells. (**a**) Staining of Rat-1 cells using monoclonal anti-pp60$^{v\text{-}src}$. (**b**) Staining of VIT cells using monoclonal anti-pp60$^{v\text{-}src}$.

of these are believed to interact with microtubules, intermediate filaments (Heuser & Kirschner, 1980) and other subcellular features.

Changes in the actin distribution in cells following viral transformation have been observed by several investigators using various microscopic techniques. Immunofluoroscence microscopy has demonstrated larger microfilament bundle networks within whole cells (Goldman *et al.*, 1975) and a reduction in the number and thickness of the bundles has been demonstrated following viral transformation by RNA viruses (Ash *et al.*, 1976, Wang & Goldberg, 1979, Boschek *et al.*, 1981) and DNA viruses (Osborn & Weber, 1975, Pollack *et al.*, 1975). However, the limited resolution of this technique does not identify the finer detail of these changes. At higher resolution, transmission electron microscopy on thin sections has demonstrated that on viral transformation actin is found either as dispersed microfilaments arranged in more loosely interwoven networks than the bundled networks of untransformed cells (Wang & Goldberg, 1976), or that fewer microfilaments are present in transformed cells and these are not distributed in bundled networks (McNutt *et al.*, 1973). Whole cell mount transmission electron microscopy has been used to demonstrate cytoskeletal networks at a higher resolution than is possible by light microscopy methods, and allows some appreciation of the three dimensional nature of the structures in the whole cell which is not possible using thin sections of tissues. The technique does require triton-extraction of cells, and this removes a substantial amount of the cytoplasmic proteins, including some cytoskeletal proteins. However, with this reservation we have used the technique to demonstrate changes in the triton-insoluble cytoskeleton to correlate with biochemical studies.

Few studies, however, have been directed towards the more dynamic aspects of these changes. The actin monomer-polymer equilibrium state in a cell cytosol compartment might play an important role in transformation, in which changes in the subcellular distribution of filamentous actin occur. Robbins *et al.* (1975) used a differential centrifugation and polyacrylamide gel scanning technique to assess the status of actin in untransformed chick fibroblasts. They found that they contained 45.6% actin in an unpolymerized form, and on transformation by RSV this monomer pool increased to 60–64%. This study therefore suggested that filamentous actin was depolymerized on transformation. Our studies, however, have demonstrated that the changes are more subtle than simply an overall shift in the monomer-polymer equilibrium in the direction of disassembly.

The aim of the present study was to investigate whether the apparent loss of stress fibres on transformation of cells represented simply a depolymerization of actin filaments *per se*, or whether the filamentous actin was reorganized at some other site(s) within the cell. We have fractionated cells into cytosol and cytoskeletal core subfractions by low speed centrifugation, a procedure which separates the larger macromolecular actin assemblies in the membrane-associated cytoskeletal core from the monomeric actin and non-bundled, non-cross-linked single actin filaments within the cytosol. Our studies on Rat-1 fibroblasts have clearly demonstrated that RSV transformation is not associated with any major change in the total amount of actin within the cells. The overall cell actin status is marginally shifted towards actin depolymerization, but the monomer–polymer equilibrium within the cytosol fraction is very significantly disturbed in the direction of disassembly and the proportion of actin in the cytoskeletal core fraction is significantly higher in the transformed cells than in the non-transformed cells. This change in the cytosol monomer–polymer equilibrium is likely to reflect depolymerization of cytosolic actin filaments and the increase in cytoskeletal core actin seen in transformed cells could indicate a new assembly of actin recruited from the cytosol monomer and/or polymer pool. Whole cell mount transmission electron microscopy have confirmed that the Triton insoluble cytoskeleton is substantially altered in its macromolecular character. The well expressed actin filament bundles seen in the Rat-1 cells appear to have been replaced by compact aggregates and submembraneous assemblies. That these aggregates and submembranous structures are largely composed of actin-containing filaments is supported by the fluorescence studies.

However, the mechanism of these changes in the character and disposition of actin following transformation is by no means understood. In this study, the *src* gene product, pp60$^{v\text{-}src}$ was shown to be located at the plasma membrane and it has been demonstrated by others to be concerned with tyrisone phosphorylation of certain cytoskeletal components (Hunter & Cooper, 1983). Almost certainly such covalent modifications would affect membrane/cytoskeletal and other protein/protein associations. In other studies the pp60$^{v\text{-}src}$ has been shown to be specifically associated with the cytoskeleton of transformed chick embryo fibroblasts (Burr *et al.*, 1980) and proteins containing phosphotyrosine revealed after RSV transformation of mouse fibroblasts have been shown to be concentrated at the ventral plasma membrane associated with adhesion plaques (Shriver & Rohrschneider, 1981). Although a direct involvement of pp60$^{v\text{-}src}$ activity with specific cytoskeletal proteins, whose functions may then be significantly altered, has not been demonstrated, the induction of tyrosine-specific phosphorylation of vinculin by pp60$^{v\text{-}src}$ has been shown. The significance of this remains unclear, however, since only ~1% of the total vinculin in the cell is so modified, and the phosphorylation does not appear to correlate in time or magnitude with any changes in microfilament redistribution or in cell morphology (Rosok & Rohrschneider, 1983). A membrane-associated 36 kDa protein which binds to non-erythroid spectrin and F-actin in a $Ca^{2+}$ dependent manner has been shown to be phosphorylated on tyrosine residues following RSV transformation, but it is not known if such a modification is of functional significance with respect to these membrane-associated structural proteins (Gerke & Weber, 1984). The observation that unphosphorylated gelsolin, a 90 kDa actin-destabilising protein known to be present in highly motile cells such as macrophages and platelets is localized in regions of cell substratum contact in RSV-transformed rat cells (Wang *et al.*, 1984) could suggest that it too may play a role in the actin polymer–monomer equilibrium shifts such as we have observed.

In conclusion, therefore, we have demonstrated that RSV transformation of Rat-1 fibroblasts is associated with cytosolic actin depolymerization, and an increase in filamentous actin in the cytoskeletal core. These changes are complex and are likely to occur as a consequence of changes in the cytosol environment or modifications to actin regulatory protein function. The shifts observed in the cytosolic actin monomer–polymer equilibrium state could play a significant role in some of the salient morphological features of transformation, and may specifically be associated with alteration in cell shape towards the more 'rounded up' morphology, as also in the decreased adhesiveness of cells. An increased incorporation of actin into a membrane-associated cytoskeletal core affecting cellular motile properties such as the invasive characteristics of malignant tumour cells would be an attractive hypothesis but further studies are required if the validity of this is to be established with any certainty.

We thank Dr J. Brugge (SUNY, Stonybrook, USA) for providing us with the monoclonal anti-pp60$^{v\text{-}src}$ and Dr P.J. Enrietto (ICRF) for supplying tumour-bearing rabbit serum.

We also thank Dr Steve Gschmeissner of the Anatomy Department, Royal College of Surgeons of England, for his help with the Electron Microscopy and Miss Heather Watson for her help in preparation and typing of the manuscript.

Finally, we wish to express our appreciation to Dr G. Poste, Vice President, Research and Development, of Smith Kline & French Ltd., Philadelphia, for kindly arranging support for some of our research costs.

### References

ABERCROMBIE, M. & DUNN, G.A. (1975). Adhesions of fibroblasts to substratum during contact inhibition observed by interference reflection microscopy. *Exp. Cell Res.*, **92**, 57.

ASH, J.F., VOGT, P.K. & SINGER, S.J. (1976). Reversion from transformed to normal phenotype by inhibition of protein synthesis in rat kidney cells infected with a temperature sensitive mutant of Rous sarcoma virus. *Proc. Natl., Acad. Sci* (*USA*), **73**, 3603.

BLIKSTAD, I., MARKEY, F., CARLSSON, L., PERSSON, T. & LINDBERG, U. (1978). Selective assay of monomeric and filamentous actin in cell extracts, using inhibition of deoxyribonuclease 1. *Cell*, **15**, 935.

BOSCHEK, C.B., JOCKUSCH, B.M., FRIIS, R.R., BACK, R., GRANDMANN, E. & BAUER, M. (1981). Early changes in the distribution and organization of microfilament proteins during cell transformation. *Cell*, **24**, 175.

BRITCH, M. & ALLEN, T. D. (1981). The effects of cytochalasin B on the cytoplasmic contractile network revealed by whole cell transmission electron microscopy. *Exp. Cell Res.*, **131**, 161.

BURR, J.G., DREYFUSS, G., PENMAN, S. & BUCHANAN, J.M. (1980). Association of the *src* gene product of Rous sarcoma virus with cytoskeletal structures of chick embryo fibroblasts. *Proc. Natl. Acad. Sci.* (*USA*), **77**, 3484.

CHISWELL, D.J., ENRIETTO, P.J., EVANS, G., QUADE, K. & WYKE, J.A. (1982). Molecular mechanisms involved in morphological variation of avian sarcoma virus-infected rat cells. *Virology* **110**, 428.

COLLETT, M.S. & ERICKSON, R.L. (1978). Protein kinase activity associated with avian sarcoma virus *src* gene product. *Proc. Natl. Acad. Sci.* (*USA*), **75**, 2021.

ERIKSON, R.L., PURCHIO, A.F., ERIKSON, E., COLLETT, M.S. & BRUGGE, J.S. (1980). Molecular events in cells transformed by Rous sarcoma virus. *J. Cell Biol.*, **87**, 319.

GEIGER (1979). A 130 K protein from chicken gizzard: its localisation at the termini of microfilament bundles in cultured chicken cells. *Cell*, **18**, 193.

GERKE, V. & WEBER, K. (1984). Identity of p36K phosphorylated upon Rous sarcoma virus transformation with a protein purified from brush borders: calcium-dependent binding to non-erythroid spectrin and F-actin. *Embo J.*, **3**, 227.

GOLDMAN, R.D., LAZARIDES, E., POLLACK, R. & WEBER, K. (1975). The use of actin antibody in the localization of actin within the microfilament bundles of mouse 3T3 cells. *Exp. Cell Res.*, **90**, 333.

GOLDMAN, R.D., YERNA, M.J. & SCHLOSS, J.A. (1976). Localization and organization of microfilaments and related proteins in normal and virus transformed cells. *J. Supramol. Struct.*, **5**, 155.

HEUSER, J.E. & KIRSCHNER, M.W. (1980). Filament organization revealed in platinum replicas of freeze-dried cytoskeletons. *J. Cell. Biol.*, **86**, 212.

HUNTER, T. & COOPER, J.A. (1983). Role of tyrosine phosphorylation in malignant transformation by viruses and in cellular growth control. *Prog. Nucleic Acid Res. Mol. Biol.*, **29**, 221.

KELLIE, S., PATEL, B., PIERCE, E.J. & CRITCHLEY, D.R. (1983). Cocapping of α-actinin with cholera toxin-ganglioside GM, complexes on lymphocyte cell membranes. *J. Cell Biol.*, **97**, 447.

MANESS, P.F., ENGESER, H., GREENBERG, M.E., O'FARRELL, M., GALL, W.E. & EDELMAN, G.M. (1979). Activities of the *src*-gene product of avian sarcoma virus. *Cold Spring Harbour Symp. Quan. Biol.* **44**, 949.

McNUTT, N.S., CULP, L.A. & BLACK, P.H. (1973). Contact-inhibited revertant cell lines isolated from SV-40 transformed cells. IV. Microfilament disruption and cell shape in untransformed, transformed and revertant Balb/c 3T3 cells. *J. Cell Biol.*, **56**, 412.

OSBORN, M. & WEBER, K. (1975). Simian virus 40 gene. A function and maintenance of transformation. *J. Virol.*, **15**, 636.

PERKINS, R.M., KELLIE, S., PATEL, B. & CRITCHLEY, D.R. (1982). Gangliosides as receptors for fibronectin? *Explt. Cell Res.*, **141**, 231.

POLLACK, R., OSBORN, M. & WEBER, K. (1975). Patterns of organization of actin and myosin and normal and transformed cultured cells. *Proc. Natl. Acad. Sci.* (*USA*), **72**, 994.

POLLARD, T.D. & WEIHING, R.R. (1974). Actin and myosin and cell movement. *CRC Crit. Rev. Biochem.*, **2**, 1.

ROBBINS, P.W., WICKUS, G.G., BRANTON, P.E. & 4 others (1975). The chick fibroblast cell surface after transformation by Rous sarcoma virus. *Cold Spring Harbor Symposium on Quant. Biol.*, **39**, 1173.

ROHRSCHNEIDER, L.R., ROSOK, M.J. & GENTRY, L.E. (1983). Molecular interaction of the *src* gene product with cellular adhesion plaques. *Prog. Nucleic Acid Res. & Mol. Biol.*, **29**, 233.

ROSOK, M.J. & ROHRSCHNEIDER, L.R. (1983). Increased phosphorylation of vinculin on tyrosine does not occur during the release of stress fibers before mitosis in normal cells. *Mol. Cell. Biol.*, **3**, 475.

RUBIN, R.W., WARREN, R.H., LUKEMAN, D.S. & CLEMENT, E. (1978). Actin content and organization in normal and transformed cells in culture. *J. Cell Biol.*, **78**, 28.

SCHRIVER, K. & ROHRSCHNEIDER, L. (1981). Organization of $pp60^{src}$ and selected cytoskeletal proteins within adhesion plaques and junctions of Rous sarcoma virus-transformed rat cells. *J. Cell Biol.*, **89**, 525.

SEFTON, B.M., HUNTER, T., BALL, E.M. & SINGER, S.J. (1981). Vinculin: A cytoskeletal target of the transforming protein of Rous sarcoma virus. *Cell* **24**, 165.

VARMUS, H.E., QUINTRELL, N. & WYKE, J. (1981). Revertants of an ASV-transformed rat cell line have lost the complete provirus or sustained mutations in *src*. *Virology*, **108**, 28.

WANG, E. & GOLDBERG, A.R. (1976). Changes in surface topography and microfilament organization upon transformation of chick embryo fibroblasts with Rous sarcoma virus. *Proc. Natl. Acad. Sci* (*USA*), **73**, 4065.

WANG, E. & GOLDBERG, A.R. (1979). Effects of the *src* gene product on microfilament and microtubule organization in avian and mammalian cells infected with the same temperature-sensitive mutant of Rous sarcoma virus. *Virology*, **92**, 201.

WANG, E., YIN, H.L., KRUEGER, J.G., CALIGUIRI, L.A. & TAMM, I. (1984). Unphosphorylated gelsolin is localized in regions of cell-substratum contact or attachment in Rous sarcoma virus-transformed rat cells. *J. Cell. Biol.*, **98**, 761.

WEISS, R., TEICH, N., VARMUS, H. & COFFIN, J. (eds.) 1982. *RNA Tumour Viruses*. Cold Spring Harbor Laboratory.

WICKUS, G., GRUENSTEIN, E., ROBBINS, P.W. & RICH, A. (1975). Decrease in membrane-associated actin of fibroblasts after transformation by Rous sarcoma virus. *Proc. Natl., Acad. Sci.* (*USA*), **79**, 746.